# Host-microbe-cancer interactions on-a-chip

**DOI:** 10.3389/fbioe.2025.1505963

**Published:** 2025-03-31

**Authors:** Mauricio G. C. Sousa, Danielle S. K. Brasino, Madeline Krieger, Duygu A. Dindar, Rebekka Duhen, Zhenzhen Zhang, Cristiane Miranda Franca, Luiz E. Bertassoni

**Affiliations:** ^1^ Knight Cancer Precision Biofabrication Hub, Knight Cancer Institute, Oregon Health & Science University, Portland, OR, United States; ^2^ Cancer Early Detection Advanced Research Center, Oregon Health & Science University, Portland, OR, United States; ^3^ Department of Biomaterial and Biomedical Sciences, School of Dentistry, Oregon Health & Science University, Portland, OR, United States; ^4^ Department of Microbiology and Molecular Genetics, Robert Larner College of Medicine at the University of Vermont, Burlington, VT, United States; ^5^ Division of Oncological Sciences, Oregon Health & Science University, Portland, OR, United States; ^6^ Department of Biomedical Engineering, School of Medicine, Oregon Health & Science University, Portland, OR, United States

**Keywords:** tumor on-a-chip, host-microbes, microbes, tissue engineering, microfluidic devices

## Abstract

The tumor microbiota has emerged as a pivotal contributor to a variety of cancers, impacting disease development, progression, and therapeutic resistance. Due to the complexity of the tumor microenvironment, reproducing the interactions between the microbes, tumor cells, and the immune system remains a great challenge for both *in vitro* and *in vivo* studies. To this end, significant progress has been made toward leveraging tumor-on-a-chip model systems to replicate critical hallmarks of the native disease *in vitro*. These microfluidic platforms offer the ability to mimic essential components of the tumor microenvironment, including controllable fluid flow conditions, manipulable extracellular matrix dynamics, and intricate 3D multi-cellular communication. The primary objective of this review is to discuss recent challenges and advances in engineering host-microbiota and tumor interactions on-a-chip. Ultimately, overcoming these obstacles will help us gain deeper insights into tumor-microbe interactions and enhance avenues for developing more effective cancer therapies.

## 1 The need for modeling host-microbiota and cancer cell interactions

Although the contribution of the microbiome to the development of cancer has long been proposed, recent evidence has demonstrated that the microbiota plays multifaceted roles in cancer development, progression, and treatment (Fu et al., 2023; Zhao et al., 2023; Battaglia et al., 2024). These recent discoveries have sparked significant interest in understanding the type, localization, mechanisms of action, and interactions of different types of microorganisms with the tumor microenvironment (TME) and its components (Lythgoe et al., 2022; Li et al., 2024). Many tumor-resident microbes (TRMi) are opportunistic, facultative anaerobes and intracellular pathogens that induce DNA damage (Espinoza and Minami, 2018; Gu X. et al., 2020), produce genotoxins (Cao et al., 2022; Joo et al., 2024), and cause inflammation (Lu et al., 2024; Zhang et al., 2024). TRMi can inactivate anti-cancer drugs and modulate the immune system, leading to poorer prognoses for therapies (Jiang et al., 2023; Sfanos, 2023; Kang et al., 2024). The recent reports of the involvement of certain microorganisms with some tumors such as colorectal cancer (Zepeda-Rivera et al., 2024) has attracted the attention of health authorities and clinicians, urging elucidation of their roles in the induction of malignant processes, dissecting intra- and extracellular effects, and clinical outcomes.

A substantial body of work has demonstrated, through comprehensive 16S rRNA analysis on several solid tumor types (Nejman et al., 2020) and spatial profiling combined with RNA sequencing (Galeano Nino et al., 2022), that TRMi interact with cancer cells, immune cells, and the extracellular matrix. This leads to the recruitment of immune cells and transcriptional changes in cancer cells and cancer-associated fibroblasts (Galeano Nino et al., 2022; Narunsky-Haziza et al., 2022; Wong-Rolle et al., 2022; Zepeda-Rivera et al., 2024). However, elucidating the exact mechanistic events linking the presence of microorganisms and activity with cancer is challenging due to the need for reliable pre-clinical models that emulate patient conditions (Rodrigues et al., 2021;Vitale et al., 2024). *In vivo* models of host-microbiota interactions remain the most reliable tools for understanding cancer biology (Ladaycia et al., 2021; Rodrigues et al., 2021; Li G. et al., 2023), and are well-established in the literature. Nevertheless, these models cannot accurately capture the extensive heterogeneity of native microorganisms observed in the human microbiome, which is shaped by unique genetic and immune composition (Aizpurua et al., 2023; Cao et al., 2024). Furthermore, traditional cell culture and co-cultures of mammalian cells and microorganisms often inadequately represent the spatial organization and ratios inherent in the tumor microenvironment (Asghar et al., 2015; Kapalczynska et al., 2018).

Alternative models such as organotypic cultures that combine engineering and biology provide a real-time simulation of critical elements of the interactions between TRMi and the TME *in vitro* (Radhakrishnan et al., 2020; Neufeld et al., 2022). These systems include 3D extracellular matrices (Yang et al., 2024), vasculature (Zervantonakis et al., 2012), and the interplay among stromal, immune, and cancer cells with microorganisms (Jouybar et al., 2024; Shin et al., 2024). Tumors-on-a-chip, for instance, emerged as attractive *in vitro* models that can tackle several challenges associated with accurately replicating the unique properties of the tumor microenvironment (Sontheimer-Phelps et al., 2019; Maulana et al., 2024). These microdevices incorporate microfluidic channels and manipulable fluid flow to model multiple hallmarks of the disease, including shear stress (Ortega Quesada et al., 2024), diverse cell types, an extracellular matrix, control of hypoxic gradients, and metabolic exchange between microorganisms and mammalian cells (Shah et al., 2016; Thacker et al., 2020; Shin et al., 2024), among other features. These platforms may enable scientists to decipher the interfaces between TRMi and different phases of cancer development by modulating patient-derived tissues in chips and capturing critical biomechanical elements of the TME.

In this review, we describe opportunities and challenges associated with using tumor-on-a-chip models to investigate interactions between the TME and TRMi. We discuss the requirements for designing chips that represent the specificities of the TME and exemplify some applications of these platforms in tumors directly known to harbor microorganisms.

## 2 Organs on-a-chip to understand host-microbe dysbiosis and its role in cancer development

**FIGURE 1:**
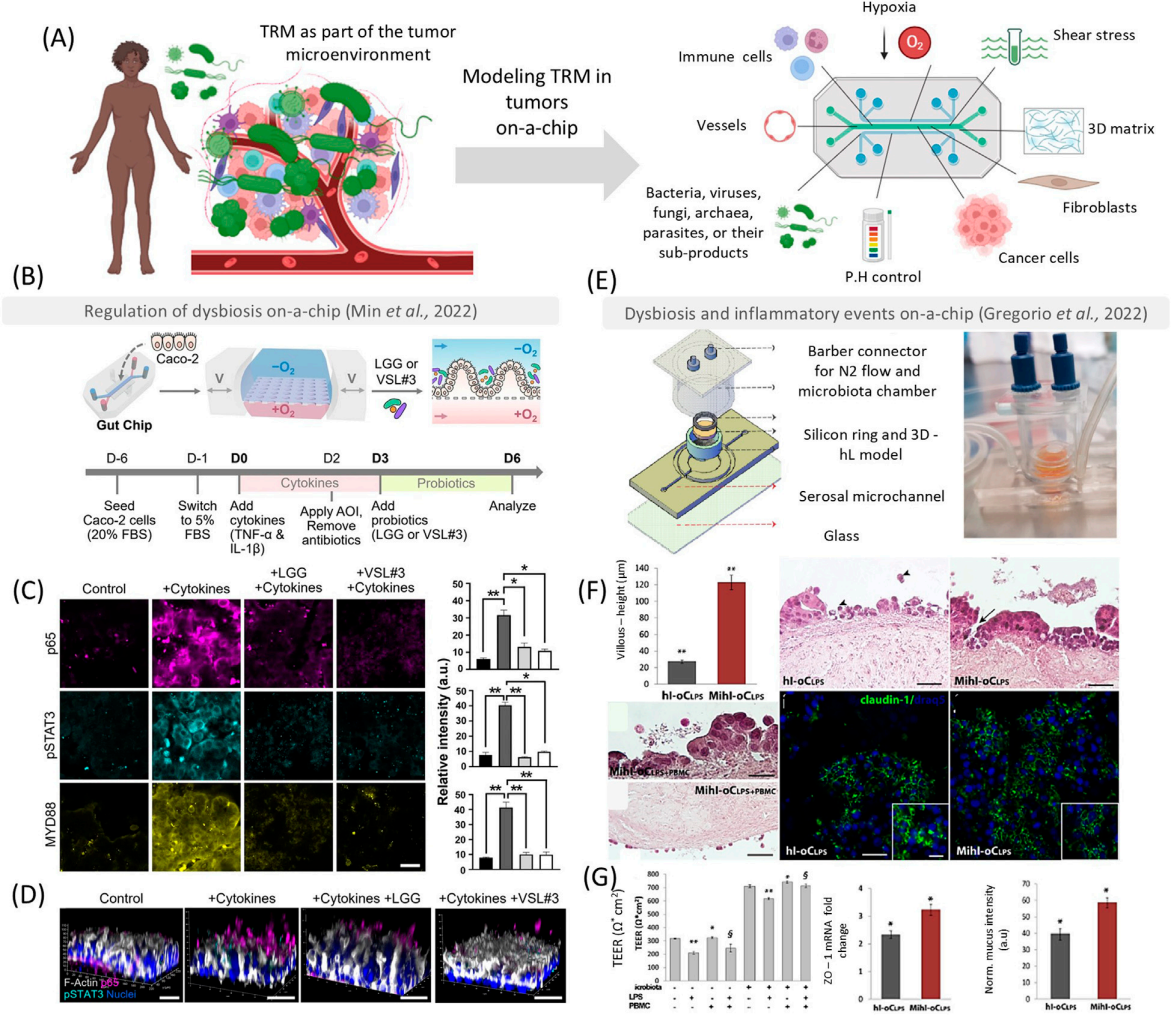
Tumor on-a-chip as platforms to emulate the host-microbe interactions in early oncogenesis and inflammation. These models can be used to emulate essential hallmarks of the tumor microenvironment, such as shear stress, hypoxia, a 3D matrix, different cell types, and interactions with several bacteria, fungi, viruses, archaea, parasites, and their sub-products **(A)**. In **(B)** reproduced from Min et al. (2022) a polydimethylsiloxane (PDMS) chip was used to understand the role of probiotics in reestablishing gut homeostasis. Chips that were treated with *Lactobacillus rhamnosus* (LLG) or a complex mixture VSL#3 could reduce inflammatory and carcinogenic signaling pathways (p65 – magenta, pSTAT3 - cyan, and MYD88) after 3 days [**(C, D)** scale bar – 50 µm]. In **(E, F)**, reproduced from de Gregorio et al. (2022), the authors developed a device that features an open central chamber accommodating a transwell membrane separating the lumen and serosal sides to emulate a human intestine on-a-chip (hi-oC) **(E)**. The authors observed that lipopolysaccharide (hl-oCLPS) would stimulate a higher inflammatory response compared to the hi-oC **(F)**, and the probiotics (Mihl-oCLPS) improved tight junction formation and mucus production **(G)**. Scales bars in **(F, G)** represent 100 µm. Parts of this figure were created with biorender.com. This figure was licensed under a common creative attribution [CC by 4.0 Spring Nature–**(B–D)** and Elsevier **(E–G)**].

Despite using microfluidic devices to study tumor-microbe interactions represents a relatively new development, the relationship between microorganisms and healthy tissues has been modeled for at least 20 years (Liu and Zhu, 2005; Kim and Sung, 2024). In the organs on-a-chip field, the gut was one of the first targets for chip modeling (Huh et al., 2013), and gut-on-a-chip devices have become very popular among researchers (Kim and Sung, 2024). These models have been improving in incorporating characteristics of native tissues and their interfaces, facilitating the growth of native-like microbial species (Ballerini et al., 2025). As a result, they have significantly enhanced the diversity and heterogeneity of microbial models, enabling the inclusion of bacteria, fungi (Srinivasan et al., 2011), viruses (Bein et al., 2021), and parasites (Nikolaev et al., 2020). Such advancements are critical for studying microbial species that may directly or indirectly contribute to dysbiosis, inflammation, and tumor establishment (Figure 1).

In the context of tumor initiation, microfluidic technologies hold significant potential to elucidate the causative mechanisms underlying the association between dysbiosis on epithelial barriers and cancer development (Poceviciute and Ismagilov, 2019). However, reproducing multispecies cultures *in vitro* presents challenges due to the complex interactions of these microorganisms on their native tissue, which occur under varying oxygen conditions (e.g., skin, colorectal, and oral cavities) and dynamic metabolic interactions (Moossavi et al., 2022). Static systems can promote microorganism overgrowth, which may overwhelm the rate of mammalian/microorganism cell interactions (Jalili-Firoozinezhad et al., 2019; Shin et al., 2024). Microfluidic devices can emulate tight junction formation, epithelial layers, mucus, and peristaltic movements related to shear stress to adopt microbial diversity and interactions with epithelial cells during early cancer development (Sibilio et al., 2024). These devices also serve as excellent platforms for studying bacterial-carcinogenic signaling pathways (Valiei et al., 2023). For instance, a PDMS chip was designed to mimic the gut, featuring multiple rows of microgut culture chambers (Lee et al., 2024). The microgut was generated using Caco cell layers in collagen gels, creating a villus structure subjected to shear stress, hypoxia (1% oxygen to facilitate bacterial growth for 1 day), and flow to support epithelial tissue formation. After 6 days, the monolayer exhibited a well-differentiated brush border, various intestinal cells (including goblet cells, enterocytes, and Paneth cells), defined tight junctions, and mucin production. The system supported the growth of both enterotoxigenic (ETBF8 and ETBF9) and non-toxigenic (NTBF) strains of *Bacteroides fragilis*. In this model, only the ETBF9 strain significantly stimulated pre-oncogenic signaling pathways, such as WNT-β-catenin and p-STAT3, compared to the microorganism-free group. Introducing *Lactobacillus rhamnosus* into the microgut significantly inhibited ETBF9 growth. These findings were previously observed only *in vivo* and demonstrate that microfluidic devices can replicate substantial biomimetic physiological and pre-carcinogenic events (Owens et al., 2021).

## 3 Modeling tripartite tumor-microbe-immune cell interactions

Another important area of exploration in recent years is the crosstalk between microbiota and the immune system in cancer (Jayakrishnan et al., 2024). Dysbiosis can lead to pathobiont proliferation and inflammation (Biragyn and Ferrucci, 2018). Opportunistic microorganisms, such as *F. nucleatum* and *Candida albicans*, proliferate under dysbiosis conditions (Gu P. et al., 2020; Li et al., 2022). Their interaction with epithelial and cancer cells triggers inflammatory cytokines (e.g., interleukins IL-6, IL-17, IL-8) and pathways (e.g., NF-kB, STAT3), promoting epithelial-to-mesenchymal transition, immune chemoattractants, and cancer cell proliferation (Narunsky-Haziza et al., 2022; Wang and Fang, 2023; Wang et al., 2023b). Additionally, microorganisms interact with the immune system in cancer through toll-like receptors (TLR2 and TLR4) and myeloid-derived suppressor cells, triggering an immunosuppressive environment (Yang et al., 2017; Wong and Yu, 2023). Some of these interactions have therapeutic consequences, especially in immunotherapies. Recent evidence has shown that *F. nucleatum* can produce succinic acid which neutralizes the effect of anti-PD1 antibodies (Jiang et al., 2023).

Combining all elements of the epithelium, the microbiome, and cancer cells together with immune cells *in vitro* is challenging. However, microfluidic devices have recapitulated some of these complexities by providing an architecture that supports immune cell infiltration, differentiation, and communication with microorganisms, which can be directly applied to the TME (Figures 1A–D). Researchers have developed a microfluidic device to understand the immune-responsive human microbiota-intestine axis (De Gregorio et al., 2022) (Figures 1E–G). This device features an open central chamber to accommodate a trans-well membrane separating the lumen and serosal sides. This chamber is housed within a poly(methyl methacrylate)-derived cap to provide N2 flow for an anaerobic luminal environment to support the growth of *L. rhamnosus* and *Bifidobacterium longum*. The microorganisms studied exhibited a protective immunoregulatory effect in the presence of lipopolysaccharide (LPS, derived from *E. coli*). Peripheral blood mononuclear cells (PBMCs) introduced to the chip produced higher levels of anti-inflammatory cytokines (such as IL-10) and lower levels of inflammatory cytokines (such as IL-2), demonstrating that the immunomodulatory role of probiotics can be recapitulated in complex models (De Gregorio et al., 2022) (Figures 1E–G).

Considering the immune system’s complexity, which requires antigen presentation and interaction with other systems, such as the circulatory and lymphatic systems, leaves several opportunities remain to explore host-microbe interactions in cancer. However, as blood and lymphatic vessels can be engineered in organs-on-a-chip, integrating multiple aspects of the microbiome, epithelial tissues, and immune cells with the vasculature, will provide robust tools to predict cancer development and progression.

## 4 Host-microbe interactions and tumors on-a-chip to understand colorectal, oral, and breast cancer

**FIGURE 2:**
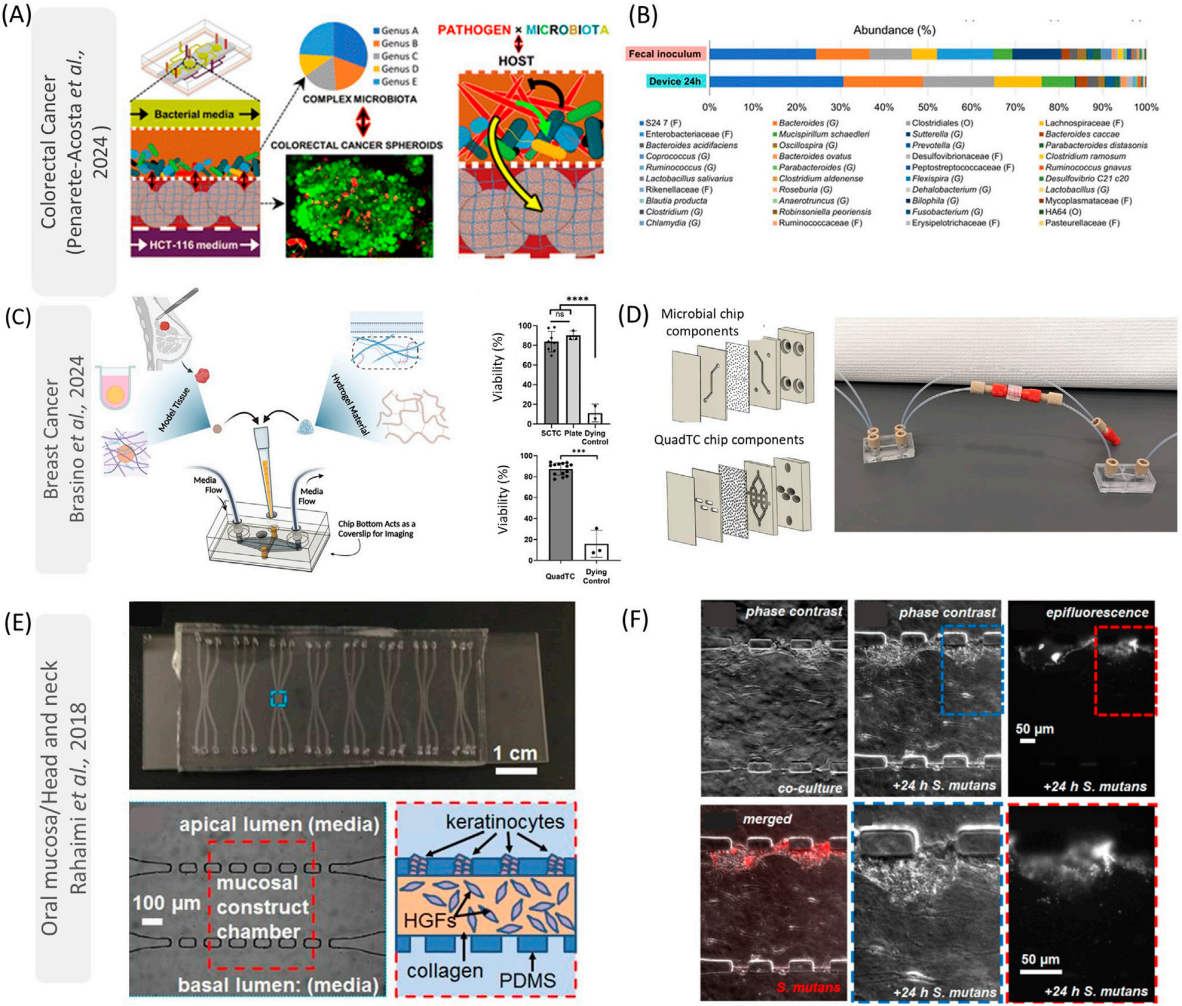
Applications of on-chip models to understand host-microbe interactions in cancer for colorectal cancer **(A, B)**, breast cancer **(C, D)**, and oral mucosa/head and neck **(E, F)**. In **(A)**, Penarete-Acosta et al. (2024) developed a colorectal tumor on-a-chip to evaluate the effect of *F. nucleatum* in modulating the microbiome. They found that this microorganism is responsible for increasing the abundance of pro-inflammatory microorganisms **(B)**. In **(C)**, reproduced from Brasino et al., developed microfluidic devices that can keep patient-derived organoid viability **(C)** while communicating with the gut microbiome **(D)**. These polycarbonate chips allow researchers to study hypoxic environments (gut) while connecting those systems with other distal tissues **(D)**. In **(E, F)**, reproduced from Rahimi et al. (2018), a PDMS device was designed to mimic the oral mucosa interaction with microorganisms. These devices have a central chamber containing gingival fibroblasts (HGFs) in collagen and a layer of keratinocytes **(E)**. Although the authors just evaluated the species *S. mutans* **(F)**, this platform can be used to study how oral pathobiont microorganisms can interact with head and neck cancers. This figure was licensed under a common creative attribution (CC by 4.0 Royal Chemical Society, Wiley and Elsevier). **(E, F)** was reproduced from [Christopher Rahimi, Benjamin Rahimi, Dominic Padova, Seyed A. Rooholghodos, Diane R. Bienek, Xiaolong Luo, Gili Kaufman, Christopher B. Raub; Oral mucosa-on-a-chip to assess layer-specific responses to bacteria and dental materials. Biomicrofluidics 1 September 2018; 12 (5): 054106. https://doi.org/10.1063/1.5048938], with the permission of AIP Publishing.

Due to the diversity and heterogeneity of microbes across various tissues, there is a compelling need to simulate their interactions within specific physiological environments in the human body (Shin et al., 2024). For instance, the gastrointestinal tract and oral cavity can be inhabited by anaerobic pathobionts (Zhao et al., 2023; Liao et al., 2024), while the mammary glands can harbor aerobic species (Tzeng et al., 2021). Furthermore, numerous uncertainties persist regarding how local and tissue-specific microbes might influence the initiation, progression, and resistance of distant tissue tumors (Fu et al., 2023). To address many of these biological questions, organ-on-a-chip technologies enable the investigation of microbe interactions with specific tissues (such as the tumor microenvironment) and their spread to other tissues (metastasis). In this section, we elucidate the applications of these advanced models in studying various cancer types, such as colorectal, breast, and head and neck cancers (Figure 2).

### 4.1 Colorectal cancer

According to the World Health Organization, colorectal cancers (CRCs) are the second leading cause of cancer-related deaths worldwide and were responsible for causing almost one million deaths in 2023 (World Health Organization, 2023a). Mounting evidence shows that our microbiota is involved in several aspects of CRC development (Lynch and Pedersen, 2016; Cui et al., 2024). Several bacterial species may play a role in the initiation or progression of GI cancer (Lotstedt et al., 2023). Gut dysbiosis can cause variations in the normal pH balance, generate genotoxins, trigger adhesion receptor interactions, induce inflammation, trigger epithelial-mesenchymal transition, chemoattract immunosuppressive cells, and produce a hypoxic environment that favors the growth of anaerobic microorganisms (Rath and Haller, 2022; Simpson et al., 2022; White and Sears, 2023). All these factors are critical to establish the tumor microenvironment bioarchitecture. Organ-on-a-chip models to study host-microbe interactions in CRC have been shown to mimic dysbiosis *in vitro*, to elucidate in real-time key signals, proteins, metabolites, and cytokines involved with CRC initiation (Udayasuryan et al., 2020).

One of the benefits of using microfluidic devices to study gut microbiota and colonocytes is that these interactions can be manipulated to provide an understanding of contact-independent mechanisms and metabolic exchanges between specific microorganisms and cancer cells in real-time (Mitrofanova et al., 2024). For example, researchers have developed a microfluidic device to mimic the non-physical interactions between *F. nucleatum*, colonocytes, and commensal microbiota (Penarete-Acosta et al., 2024). The polydimethylsiloxane (PDMS) microfluidic device used in this study had four microfluidic layers: two in the middle for mammalian cells and bacteria, and one top and bottom layer for media. These layers are each separated by porous polyester membranes to prevent physical interaction between the bacteria and colonocytes (HCT116 cell line). The colonocytes were cultivated in Matrigel, and the chip was then transferred to an anaerobic chamber to mimic the hypoxic colorectal environment. In this model, *F. nucleatum* stimulated genes related to inflammation/cell signaling (such as IL-8 and VEGF), and epithelial-mesenchymal transition (SNAI2). Additionally, microorganisms from the murine fecal microbiota were seeded directly into the chip model and used to study the impact of *F. nucleatum* on microbial composition. Although a decrease in mammalian cellular viability was observed over 24 h due to the challenges in cultivating mammalian cells in an acidic and anaerobic environment enriched with microorganism metabolites, the presence of *F. nucleatum* appeared to modulate the microbiome, upregulating the presence of some genera such as *Suterella* and *Clostridium* (Penarete-Acosta et al., 2024) (Figures 2A, B).

The potential use of microfluidic devices to explore the gut microbiome and colonocytes is promising; however, maintaining the viability of different microbial species and mammalian cells in the same device is still a major challenge. Furthermore, incorporating integral aspects of the innate and adaptive immune system such as neutrophils, macrophages, and T cells will aid in the understanding of how dysbiosis impacts tumor microenvironment initiation and establishment. A recent study demonstrated the development of human intestinal immune-organoids to recapitulate and investigate drug-induced intestinal inflammation (Recaldin et al., 2024). Thus, combining patient-derived colorectal organoids, immune cells, fecal microbiota, we can envision to establish complex microphysiological systems that could serve as personalized diagnostics, leveraging live biopsies that can be cultivated, manipulated and treated in real-time.

### 4.2 Breast cancer

Breast cancers (BC) are heterogeneous diseases derived from the ductal epithelium of mammary tissue (Danenberg et al., 2022) and are the leading type of cancer in women (Siegel et al., 2024). BC are composed of a microenvironment rich in myoepithelial cells and stromal cells, including immune cells, fibroblasts, and adipocytes condensed in an extracellular matrix (Chung et al., 2017; World Health Organization, 2023b). Recent studies have identified several breast bacterial taxa that appear to be associated with BC, such as the genera *Staphylococcus, Enterococcus, and Streptococcus* (Banerjee et al., 2021; Tzeng et al., 2021). Healthy versus cancer tissues have demonstrated differences in microbiome composition, with malignant environments presenting a higher composition of facultative anaerobic microorganisms (Fu et al., 2022). The presence of intratumoral bacteria is also thought to be critical to metastasis, regulating the cellular resistance to fluid stress by reorganizing the cytoskeleton (Fu et al., 2022).

The metabolic and hypoxic environment in breast cancer dictates a challenging task to be reproduced *in vitro.* Furthermore, although several pieces of evidence illustrate the role of gut microorganisms affecting estrogen metabolism and the immune system in breast cancer (Urbaniak et al., 2016; Plaza-Diaz et al., 2019; Heath et al., 2024), the communication between these distant tissues is not recapitulated in current tissue culture models. Microfluidic models may enrich our understanding of the dynamics linked with intratumoral or gut microbiota and breast cancer. For instance, Brasino et al. (2024) fabricated chips with gas-impermeable polycarbonate without tapes or gaskets. The authors cultivated colon epithelial cells (Caco2) for 6 days to form a villus-like structure followed by introducing *Lactobacillus plantarum* or a breast cancer-associated bacterium, *Blautia coccoides*. It was demonstrated that bacteria could be found on villus surfaces following 2 days of co-culture while maintaining epithelial viability, showing the functionality of interactions between facultative or obligate anaerobes and the colon-like layer structure. The authors also demonstrated that cultivating breast tumor spheroids (MCF7 cells cultivated in collagen) in microfluidics could provide a platform to elucidate the interactions between the gut microbiota and breast tumors (Figures 2C, D). By directing the flow within the chip from the microbial compartment (gut) to the breast tumor spheroid, the researchers facilitated metabolite exchange between gut microbes and the tumor. Interestingly, spheroids downstream of gut chips cultured with Estradiol-Glow-supplemented media exhibited significantly elevated expression of GREB1, an estrogen-responsive gene transcript, compared to those treated with the vehicle control (Brasino et al., 2024). These findings provide novel insights into the bidirectional relationship between gut microbes and estrogen signaling, highlighting potential mechanisms of microbial influence on tumor biology.

The possibility of creating microfluidic devices replicating hallmarks of ductal epithelium and its connection with vasculature might direct the knowledge of metastatic niches and metabolic interactions of microorganisms with distant tissues, such as the gut-breast axis. More studies are needed however to evaluate the role of specific extracellular microorganisms and the immune system in breast cancer progression and metastasis.

### 4.3 Head and neck cancers

Head and Neck Cancers (HNCs) are the sixth most common cancer and are known for their high heterogeneity and disparity in therapeutic conduct (World Health Organization, 2024). Despite some progress in understanding the mechanism of this disease over time, some hallmarks of its complex microenvironment still need to be explored, mainly due to the need for more advanced *in vitro* models (Almela et al., 2021). The oral cavity is a unique environment hosting more than 700 species of microorganisms with a spectrum of oxygen requirements, interfaces with soft and hard tissues, and connection with the gastrointestinal tract (Baker et al., 2023). Within this diverse milieu, key microorganisms such as *F. nucleatum, Prevotella intermedia, Porphiromonas gingivalis, C. albicans*, and human papillomavirus (HPV), among others correlate with HNC initiation, progression, and treatment resistance (Yu et al., 2017; Galeano Nino et al., 2022; Wang et al., 2023a; Wang et al., 2023c). With the increasing number of HPV + HNCs worldwide (Califano et al., 2023; Ramos da Silva et al., 2023), developing biological systems to reproduce HNCs *in vitro*, including host-microbe interactions accurately, remains a substantial barrier to further research, especially in early cancers. This challenge partly arises because some of the TRMis are opportunistic, and their pre-cancer mechanisms are still poorly understood. Furthermore, a critical barrier persists in elucidating how slight alterations in dysbiosis, and the associated metabolites may influence oncogenesis in cancer development. These phenomena raise numerous unanswered epigenetic questions that require further investigation.

The oral mucosa is the main barrier in the oral cavity and is constantly in contact with oral saliva and several microorganisms in the oral cavity (Baker et al., 2024). The host-microbe interactions happen mainly in the epithelial layer, where epithelial cells participate directly in the inflammatory response to dysbiosis and can undergo epithelial-to-mesenchymal transition which progresses the disease (Cai et al., 2024). It has been reported that some microorganisms involved with periodontal plaques such as *F. nucleatum* and *P. gingivalis*, participate in the tumor microenvironment, especially in immunosuppressive areas (Galeano Nino et al., 2022). Although the mechanisms involved with oral microorganisms and oncogenesis are still to be fully understood, microfluidic devices are essential tools to recapitulate those phenomena, especially by providing organotypic functions and interactions with microbes and their subproducts, while being visualized under the microscope for long-term cultures (da Costa Sousa et al., 2024). In this direction, several models of gingiva and oral mucosa have been developed in the literature, especially to evaluate biomaterials-soft tissue interactions, and response to radiation (Tabatabaei et al., 2020; Ly et al., 2021; Ly et al., 2022; Muniraj et al., 2023) (Figures 2E, F). For example, Muniraj et al. (2023) developed a microphysiological model of gingiva on-a-chip. This microfluidic device was prepared by thermal bonding of polymethyl methacrylate sheets with different thicknesses. Both lower (metabolic waste) and upper chambers were connected to independent inlets and outlets. The main chamber was used to cultivate gingival equivalents, separated from the lower chamber by a polycarbonate porous membrane. The authors observed that after 3 weeks of culture, the samples did not contract and maintained the epithelial structure presenting multi-layered, ortho-keratinized, squamous epithelium over a cellular lamina propria. Furthermore, the engineered tissue presented a high expression of filaggrin, loricrin (epithelial barrier), collagen-IV (basement membrane), and laminin-V (epithelial junction). Also, it was observed that the gingiva-on-a-chip could respond to mucosal irritation (alcohol mouthwash), providing outputs of cell metabolic activity similar to those obtained using gingiva inserts in MTT and LDH tests (Muniraj et al., 2023). In another investigation, Ly et al. (2022) developed mucositis on-a-chip using PDMS with 3 channels. The central channel was used to create three-layer gingival keratinocytes (GIE), human gingival fibroblasts (HGF), and human dermal microvascular endothelial cells (HMEC) in collagen that generated a mucosa-like tissue that could be cultivated for 21 days. The model was used to emulate the mucositis induced by cisplatin and radiation, which could be observed especially on day 12 of treatment, by the higher release of LDH and cellular viability (Ly et al., 2022). Although the authors did not evaluate the effect of the microbiota in mucositis development, this platform opens several opportunities to study host-microbe interactions and oral cancer on-a-chip.

Considering the complexity and diversity of HNCs, future models should integrate aspects of the saliva, oral epithelium, and other microorganisms that have been associated with oral cancers, such as the fungal genus *Candida* (Wang et al., 2023a), and HPV (Sathish et al., 2014). Additionally, these devices could be used to simulate the interactions between alcohol and smoking, both of which are known to modulate oral dysbiosis and cancer progression (Benam et al., 2016). By including immune components such as tertiary lymphoid structures (Li H. et al., 2023) these systems might also provide an advanced understanding of the dynamics of inflammatory stages, adaptive responses, and their effect on immunotherapies.

## 5 Concluding remarks and future perspectives

The microbiome is relevant to most, if not all, cancers, beyond those outlined here, including malignancies of the skin, lung, urogenital system, and bone marrow. Therefore, there is a need to develop models that can mimic the physiology of these tissues and their connections with the microbiome (Ramirez-Labrada et al., 2020; Martin et al., 2022; Voigt et al., 2022; Woerner et al., 2022). Despite documentation of interactions between the microbiome and cancer development, many opportunities remain to further explore the potential of organ on-a-chip modeling within the tumor microenvironment, such as their mechanistic interactions with the immune system in the TME, immunosuppression, metastasis, and immunotherapies. Furthermore, the connection of tissue-specific microbiota with distant cancers and metastasis should be explored, primarily because the gut microbiome has been correlated with metastasis to several tissues (Fu et al., 2023).

While the outlook for host-microbes on-a-chip models in cancer studies is promising, several challenges are still to be overcome. Current models often struggle to capture the full complexity of the microbiome, including the diversity and density of microorganisms that accurately reflect local and systemic niches. Variability in environmental factors such as temperature, oxygen levels, growth media, and shear stress further complicates the standardization of experimental conditions, reducing reproducibility and comparability across studies. Furthermore, achieving the precise organization of tissues, including accurate cell-cell interactions and extracellular matrix composition, remains a significant technical challenge. Finally, the integration of advanced technologies, such as artificial intelligence and high-throughput systems, is still in its early stages, limiting the predictive power and scalability of these platforms.

Overcoming these challenges will require a multi-disciplinary approach that integrates expertise in tissue engineering, microbiology, and computational sciences. Addressing the need for microbiome diversity, optimizing growth conditions for heterogeneous microorganisms, and refining tissue engineering techniques are essential steps toward enhancing the fidelity and translational of these models. Additionally, incorporating cutting-edge technologies will not only improve data analysis but also enable the development of more robust and scalable systems. Once these limitations are addressed, organ-on-a-chip models have the potential to transform our understanding of microorganisms in cancer and develop specific immune and microbiological therapies. These platforms could serve as “live biopsies” or personalized tissue avatars, allowing clinicians to evaluate patient-specific responses to treatments in real-time or to assess microbiota profiles and correlate them with disease progression. Also, these platforms can be used to test engineered microorganisms by synthetic biology to compete with TRMis and attract, polarize, and stimulate tumor-killing immune cells. Such applications will generate several opportunities for more precise therapeutic interventions, ultimately improving treatment outcomes and enhancing the quality of life for cancer patients.

In conclusion, while the path to fully realizing the potential of organ-on-a-chip models in microbes-cancer research is linked with technical and biological challenges, their promise opens multiple doors to answer unique biological questions. These innovative platforms represent a significant step forward in understanding and treating cancer through a microbiome-informed perspective, offering a future where precision medicine can address the complexities of cancer biology accurately.
